# Clinicians’ perceived value and demographic factors that predict the utilisation of patient reported outcome measures for low back pain amongst chiropractors in Australia

**DOI:** 10.1186/s12998-021-00399-w

**Published:** 2021-11-01

**Authors:** Natalie Clohesy, Anthony Schneiders, Gaery Barbery, Steven Obst

**Affiliations:** 1 College of Health Sciences, School of Health, Medical and Applied Sciences, Central Queensland University, Branyan, QLD Australia; 2 School of Applied Psychology, Griffith University, South Brisbane, QLD Australia

**Keywords:** Outcome measures, Rehabilitation, Back pain, Allied health, Clinical practice

## Abstract

**Background:**

Factors that influence utilisation rates of patient reported outcome measures (PROMs) for low back pain (LBP) within the chiropractic profession of Australia are currently unknown. This study aimed to examine whether factors, including age, sex, experience level, clinical title (principal vs associate), or a clinicians’ perceived value of PROMs, are predictive of the frequency and/or type of PROMs used by chiropractors in the management of LBP.

**Methods:**

A cross sectional online survey was distributed to members of the Chiropractic Association of Australia (CAA now known as Australian Chiropractors Association-ACA) and Chiropractic Australia (CA). 3,014 CAA members and 930 CA members were invited to participate totaling 3,944, only respondents that were using PROMs were included in the analysis (n = 370). Ordinal logistic regression was used to examine associations between clinician demographics and perceived value of PROMs, and the frequency of pain, health, and functional patient reported outcome measure (PROM) usage by chiropractors.

**Results:**

Principal chiropractors were more likely (Wald = 4.101, p = 0.04, OR = 1.4 (1.0–2.1)) than associate chiropractors to frequently use pain-related PROMs for the management of patients with LBP. The remaining demographic factors (age, sex, and experience level) were not associated with the frequency of PROM usage; nor were the perceived value clinicians place on PROMs in clinical practice.

**Conclusion:**

Principal chiropractors were more likely to frequently use pain-related PROMs for the management of patients with LBP when compared to associate chiropractors. Demographic factors, appear to have little influence on PROM usage. While chiropractors place high value on PROMs, these beliefs are not associated with increased frequency of PROM usage for the management of LBP.

## Introduction

Patient reported outcome measures (PROMs) are tools used to measure a health status or condition from the perspective of the patient [1] and include validated questionnaires and survey instruments [2].One method of classifying PROMs can be using these five major categories: 1) health-related quality of life; 2) functional status; 3) symptoms and symptom burden (e.g., pain); 4) health behaviours; and 5) patient’s health care experience [3–5]. PROMs are used to monitor patient progress, guide clinical decision making, and benchmark treatment outcomes [5–8]. Routine use of PROMs is associated with increased therapist-patient communication [9], improved symptom control, and overall patient satisfaction [10, 11].

PROMs are recommended in clinical guidelines for a range of health disciplines and patient populations but have been most widely developed and adopted in the physiotherapy management of LBP [10, 12, 13]. A survey of New Zealand physiotherapists found that 40% of the respondents had used PROMs for LBP in the past 6 months, with the Visual Analogue Scale and Oswestry Disability Index the most commonly used PROMs [14]. Similarly, around half of physical therapists in the United States of America routinely use PROMs in the management of LBP [15]. A more recent study by Brinkman et. al., [13] in the United Kingdom revealed that 72% of physiotherapists and 71% of physiotherapy practices routinely used PROMs, the most common of which were the Numeric Rating Scale, the Patient‐Specific Functional Scale, and the Quebec Back Pain Disability Scale. Together, these studies suggest that PROMs are becoming more frequently used by physiotherapists for the management of LBP, and while difficult to determine, may relate to improved professional support and/or access to educational initiatives and resources that encourage mandatory reporting of patient outcomes. Furthermore, other factors such as the age, gender, and experience level of the clinician, including their level of educational training and attitude towards PROMs, may also influence utilisation.

A recent study of Dutch physiotherapists examined what factors influence the utilisation of PROMs in physiotherapy private practice [16]. The study showed that the age and attitude of the clinician, context, and whether the practice was certified, were weakly associated (p < 0.2) with self-reported use of PROMs. Conversely, access to electronic health record systems that enable PROM collection, and greater knowledge of PROMs, were both associated with increased PROM usage by physiotherapists [16]. Similar findings were reported in a study of New Zealand physiotherapists which found they were more likely to use PROMs if they had a master’s level qualification or equivalent and prior knowledge of the PROMs [14]. In contrast, the perceived value of outcomes measures for individuals and the ability to choose the appropriate measure, were not predictors of PROM usage [14]. While these results are important in the context of physiotherapy practice, it is not known whether factors that predict PROM use by physiotherapists, apply to other healthcare providers, such as chiropractors; as no data has previously been available.

Research on PROM utilisation within the chiropractic profession is emerging. A survey of chiropractors in Canada reported that 85% and 89% routinely used a health-related and pain-related PROM, respectively, at every initial consultation [17]. Similarly, a more recent survey of Australian chiropractors found that 72.5% of respondents use some form of PROM for all patients with LBP, with pain-related PROMs (e.g. Visual Analogue Scale) more frequently used compared to functional (e.g. Fear Avoidance Behaviour Questionnaire) or health-related PROMs (e.g. Functional Rating Index)[6]. Identifying the type and strength of the factors that predict PROM usage is an important first step when designing targeted interventions that aim to address the barriers to PROM use by chiropractors [6]. Therefore, the aim of this study was to examine whether factors, including age, sex, experience level, clinical title (principal vs associate) or a clinicians’ perceived value of PROMs, are predictive of the frequency and/or type of PROMs used by chiropractors in the management of LBP.

## Methods

### Study design

This study used survey data collected as part of a previously published study [6]. A detailed description of the online survey methodology can be found in Clohesy et al., 2018 [6]. The online survey was distributed to members of the Chiropractors Association of Australia (CAA) and Chiropractic Australia (CA) who were registered with the Australian Health Practitioner Regulation Agency (Aphra). A total of 3,944 surveys were sent to eligible participants.

### Data collection

The original survey included 25 items that aimed to establish the type and frequency of PROM usage in LBP, and any potential barriers and facilitators to their use. For the current study, only surveys from respondents that answered, ‘Yes’, to the question “Do you use PROMs in practice?” were included in the analysis (n = 370). From these surveys the following demographic variables were obtained: 1) sex (male or female); 2) years in practice; and 3) clinical title (principal or associate). The clinical title ‘associate’ refers to an employee or contractor who works within a private practice, whereas ‘principal’ chiropractor refers to the senior practitioner, usually the clinic or practice owner.

In addition, the perceived value that PROMs had on respondents’ clinical practice were obtained using three questions scored on a 5-point Likert scale (Table 1). Therefore, the term “value” within this manuscript refers to the findings from three questions within the survey regarding the importance of PROMs to practitioners, to patients and the influence of PROMs on treatment plans.

**Table 1:** Summary of response data on the importance of PROMs in clinical practice

	Very unimportant count (%)	Unimportant count (%)	Neutral count (%)	Somewhat important count (%)	Very Important *count (%)*
Importance to the practitioner (n = 312)	3 (1.0%)	7 (2.2%)	29 (9.3%)	136 (43.6%)	137 (43.9%)
Importance to the patient (n = 310)	4 (1.3%)	21 (6.8%)	48 (15.5%)	144 (46.5%)	93 (30.0%)
Influence on treatment plans (n = 302)	9 (3.0%)	22 (7.3%)	34 (11.3%)	174 (57.6%)	63 (20.9%)

The type and frequency of PROMs used was obtained by asking the question “Which < PROM name > for LBP do you use, and how frequently?”. In the original survey this question had 8 response options: each visit, initial, weekly, every 3–6 visits, monthly, every 6–9 visits, every 9–12 visits, annually or never. For the current study, these responses were recoded into a 5-point Likert scale to describe the frequency of PROM usage (Table 2). These data were then summed for each PROM classification (pain-PROM, functional-PROM, health-PROM,) and were then recoded using the same 5-point Likert scale in Table 2. Pain-PROMs included the VAS, Pain diagram, Numeric rating scale, and the McGill Pain Questionnaire. Functional PROMs included the Oswestry Disability Index, Roland Morris Questionnaire, Quebec Back Pain Disability Scale, Functional Rating Index, Bournemouth Questionnaire, and the Fear Avoidance Beliefs Questionnaire. Health PROMs included the Short Form Health Survey 36, RAND Short Form Health Survey, Dartmouth Cooperative Functional Assessment Charts, and the Health-Status Questionnaire.

**Table 2:** Summary of response data on the frequency of PROM usage for each PROM category

	Never count (%)	Rarely count (%)	Sometimes count (%)	Often count (%)	Always count (%)
Pain-PROMs	205 (40.0%)	58 (11.3%)	121 (23.6%)	115 (22.5%)	13 (2.5%)
Health-PROMs	293 (57.2%)	178 (34.8%)	35 (6.8%)	6 (1.2%)	0 (0%)
Functional-PROMs	359 (70.1%)	130 (25.4%)	16 (3.1%)	7 (1.4%)	0 (0%)

### Statistical analysis

Participant characteristics and question frequency response data are expressed using descriptive statistics. Univariate ordered logistic regressions with proportional odds were used to determine which of the seven independent variables (sex, age, years of practice, clinical title, importance to practitioner, importance to patient, importance to treatment) were associated with the frequency of PROM use (never, rarely, sometimes, often, always) for each category (pain-PROM, functional-PROM, health-PROM). Multivariate ordinal logistic regressions were planned when two or more variables with p < 0.2 were found from the univariate analyses. Regression coefficients were expressed as odds ratios (OR) with 95% confidence intervals (95% CI) to indicate the strength of the explanatory variable to the dependent variable. The Wald Chi-Square statistic (Wald χ^2^) was included to assess the statistical significance of the explanatory variables in the statistical model, testing the null hypothesis that the model coefficients are equal to zero. All data were analysed using SPSS software version 22, with statistical significance set at p ≤ 0.05.

## Results

A total of 370 responses were included in the analysis. The number of years of clinical experience ranged from 1 to 50 with a mean (SD) of 15.9 years (11.2). The frequency distribution (%) of age ranges (years) were: 20–29 (16.8%), 30–39 (31.1%), 40–49 (24.9%), 50–59 (16.2%), 60–69 (9.2%), and 70 + (1.9%).

Response data for the importance of PROMs to the practitioner and patient, and influence on treatment, are presented in Table 1. In summary, 87.3% of respondents reported that PROMs were either ‘very important’ or ‘somewhat important’ for the practitioner, with only 3.2% reporting that PROMs were either ‘unimportant’ or ‘very unimportant’ for the practitioner. Similarly, most respondents (76.5%) reported that PROMs were either ‘very important’ or ‘somewhat important’ for the patient, with only 8.1% reporting that PROMs were either ‘unimportant’ or ‘very unimportant’ for the patient. Approximately 78.5% of respondents reported that PROMs were either ‘very important’ or ‘somewhat important’ for treatment, with 10.3% reporting PROMs as either ‘unimportant’ or ‘very unimportant’ to treatment.

A summary of the response data for the frequency of PROM usage for each category is presented in Table 2. Most respondents either ‘never’ (39.2%) or ‘rarely’ (13.5%) reported using pain-PROMs, with only 21.9% and 2.2% reporting they use these PROMs ‘often’ or ‘always’, respectively. Similarly, most respondents ‘never’ (58.1%) or ‘rarely’ (33.8%) reported using functional-PROMs, with only 7.0% and 1.1% using these PROMs ‘sometimes’ or ‘often’, respectively. Health-PROMs were either ‘never’ (68.6%) or ‘rarely’ (27.0%) used by most respondents, with only 1.6% indicating they ‘often’ use health-PROMs.

The results of the univariate logistic regressions are summarized in Table 3. The results indicate that except for clinical title, no predictor variable had a significant influence on the frequency of PROM usage. Principal chiropractors were 1.4 times more likely (Wald χ^2^ = 4.101, p = 0.04, OR = 1.4 (1.0–2.1)) to more frequently use pain-PROMs, compared to associate chiropractors. There was no influence of clinical title on the frequency of health-PROM or functional-PROM use. Similarly, there were no associations between PROM usage and the importance of PROMS to the practitioner or patient, or whether they influence treatment. As only one univariate association had a p-value < 0.2, no multivariate ordinal regressions were performed.

**Table 3:** Relationships between individual predictor variables and frequency of PROM usage for each PROM category

Predictor variable	Pain-PROMs				Functional-PROMs				Health-PROMs			
	OR (95%CI)	Wald χ^2^	df	p	OR (95%CI)	Wald χ^2^	df	p	OR (95%CI)	Wald χ^2^	df	p
Sex (female or male)	1.24 (0.89–1.74)	1.633	1	0.20	0.83 (0.58–1.19)	1.027	1	0.31	1.03 (0.69–1.53)	0.019	1	0.89
Age range	1.05 (0.93–1.19)	0.696	1	0.40	1.08 (0.94–1.23)	1.238	1	0.27	1.06 (0.92–1.22)	0.592	1	0.44
Associate or principal/owner	**0.69 (0.48–0.99)**	**4.101**	**1**	**0.04**	0.94 (0.63–1.41)	0.08	1	0.78	1.09 (0.71–1.68)	0.153	1	0.67
Years in practice	1.01 (0.99–1.02)	0.633	1	0.43	1.01 (0.99–1.02)	0.903	1	0.34	1.01 (0.99–1.03)	1.091	1	0.30
Importance to the practitioner	1.06 (0.82–1.36)	0.209	1	0.65	1.18 (0.89–1.55)	1.285	1	0.26	1.03 (0.76–1.38)	0.029	1	0.86
Importance to the patient	1.05 (0.84–1.31)	0.199	1	0.66	0.94 (0.74–1.19)	0.266	1	0.61	1.05 (0.81–1.36)	0.115	1	0.74
Influence on treatment plans	0.99 (0.79–1.23)	0.008	1	0.93	1.00 (0.79–1.27)	0.000	1	0.99	1.04 (0.80–1.35)	0.091	1	0.76

Bold values to highlight the significant finding

OR—odds ratio, 95%CI—95% confidence interval, Wald χ2—Wald Chi Square statistic, df-degrees of freedom

## Discussion

The aim of this study was to examine whether clinician demographics and/or perceived value of PROMs were associated with the frequency of PROM usage by chiropractors when managing patients with LBP. Although all survey respondent stated they use PROMs for patients with LBP, the data indicate utilisation rates were generally low, with most respondents reporting they either ‘never’ or ‘rarely’ use pain, health, or functional PROMs. Not surprisingly there were no consistent or strong predictors of increased PROM usage for any of the dependent variables. Our broad hypothesis that the value clinicians place on PROMs would influence the frequency of PROM usage was therefore not supported, despite most respondents reporting PROMs were either ‘important’ or ‘very important’ to the practitioner, patient, or when planning treatment. We did, however, find some evidence that clinical title influenced the frequency of PROM usage, with principal chiropractors more likely to use pain-PROMs more frequently than associate chiropractors.

### Influence of clinician demographic factors on PROM utilisation

Other than clinical title (associate or principal), none of the demographic factors examined had a significant influence on the frequency of PROM utilisation by chiropractors for the management of LBP. Our findings are broadly consistent with research in physiotherapists and suggest that clinician demographics have little influence on PROM usage. Age was not a significant predictor of the frequency of PROM usage. Although the mean odds ratios for age were above 1.0 for each PROM category, none reached the level of significance. Similar findings have been reported in physiotherapy [14, 18]. For example, Meerhoff et al., [16] using a combination of univariate and multivariate ordinal logistic regression found that while age was weakly associated with self-reported PROM usage by physiotherapists in the univariate analysis, it was not a significant predictor in the multivariate analysis. Similarly, a large study (n = 2916) of Dutch physiotherapists found there was no difference in age between PROM users and non-users for LBP [13]. Furthermore, and consistent with previous research [16], we found no association between experience level and the frequency of PROM usage. One might expect that more recent graduates, compared to older graduates, may be exposed to more evidence-based curriculum about PROMs as they become more validated and may therefore be more likely to routinely use PROMs, however this was not supported by the current analyses. Finally, sex of the clinician was not associated with the frequency of PROM usage. Our findings are again consistent with research in physiotherapy [16], and together suggest that sex is not a significant predictor of PROM usage by either chiropractors or physiotherapists in the management of LBP, nor does it appear to influence the success of educational packages aimed at increasing PROM usage by physiotherapists in the management of LBP [18]. Taken together these results suggest that interventions aimed at increasing the rate of PROM usage by clinicians in the management of LBP should not preferentially target clinicians based on either their age, experience level, or sex.

We assessed whether clinical title (‘associate’ or ‘principal’) may influence the frequency of PROM usage by chiropractor on the basis that principal chiropractors may have more clinical experience and familiarity with PROMs, and therefore would be more likely to routinely use them in clinical practice. Clinical titles are more commonly used within the chiropractic profession, with ‘associate’ chiropractor referring to an employee or contractor who works within a private practice, whereas ‘principal’ chiropractor refers to the senior practitioner, usually the clinic or practice owner. Hence, there is no literature in other health professions to directly compare our results. We found that principal chiropractors were 1.4 times more likely to more frequently, use pain-PROMs compared to associate chiropractors. This finding cannot be generalised to other types of PROMs as clinical title was not a significant predictor of either functional- or health-PROM usage. While difficult to determine, this may reflect the senior role the principal chiropractors have in guiding clinic policy and procedures, and their role as a mentor and role model for associate chiropractors. Hence, it is possible that principal chiropractors may place higher value on the importance of PROM for the management of LBP, compared to associate chiropractors, and more frequently implement PROMs in clinical practice; perhaps to encourage their use by associate clinicians.

### Influence of perceived value of PROMs on PROM utilisation

It was assumed that a chiropractor’s perceived value of PROMs would influence how frequently they would them, such as higher levels of importance placed on PROMs would be associated with higher rates of PROM usage. In contrast, we found no clear and consistent evidence that PROM utilisation rates are influenced by the value that clinicians place on PROMs for either the clinician, patient, or when planning patient management. This was despite the finding most respondents reporting that PROMs are ‘somewhat important’, or ‘very important’, for the practitioner, patient, and when planning treatment. The disconnect between the perceived value of PROMs and their usage in chiropractic practice appears to contrast research in other allied healthcare professions (e.g., physiotherapy), whereby a bi-directional relationship between a clinicians perceived values and use of outcome measurement has been found, such that a lack of perceived value was associated with decreased likelihood, and vice versa for greater perceived value [14, 19–21]. Furthermore, a more recent study identified that physiotherapists who had a positive attitude towards PROMs were more likely to use PROMs as electronic health records when compared to those who had a less favorable attitude [16]. In contrast, we did not find an association between perceived value of PROMs and their utilisation in clinical practice.

While difficult to determine, the relatively low utilisation rates of PROMs in the current study may have prevented strong association being observed using ordinal data (i.e., 5-point Likert scale), as opposed to the nominal data (i.e., user vs non-user) used in the physiotherapy studies. Nevertheless, our results suggest that while most chiropractors have a favourable view towards PROMs for the management of LBP, and that is consistent with other allied health professions [22], very few are implementing them in routine clinical practice. Other internal factors, such as a lack of knowledge of available PROMs, and how and when to use them, may therefore be a more important intrinsic barrier to overcome, compared to their perceived value [6]. The findings from this study was specific to the chiropractic profession and the clinical roles of chiropractors, the implications of this study may be of interest for future researcher amongst other health professionals.

### Implications and future directions

The findings from this study have implications on future research in this field, which could include developing education strategies aimed at increasing rates of PROM usage by chiropractors, with an initial emphasis on developing knowledge and improving ease of access. Designing education packages that promote PROM knowledge and behavioral change, rather than beliefs, might be effective at improving the rates of PROM usage in chiropractic practice and may be transferrable to other allied health professions.

Furthermore, based on the findings of Copeland et al. 2008 [14], whereby holding a master’s-level qualification was associated with use of PROMs in clinical practice, future research could investigate whether education level also influence usage amongst chiropractors. Future studies should also include a more expansive set of independent variables, based on existing literature in other health professions, to fully understand what factors drive PROM usage by chiropractors for the management of the LBP. Inclusion of PROMs for other common musculoskeletal conditions, such as neck pain, is also warranted.

### Limitations

This study has several limitations. First, online surveys even when directed towards target populations such as registered health professionals [22] are susceptible to low response rates and potential selection bias. However, this survey yielded a good response rate with demographic data that is consistent with workforce data (6), and therefore the sample was likely representative of the chiropractic community. Second, we did not include factors such as educational level and/or knowledge of PROMs which are known to be associated with PROM usage in physiotherapists, as suggested in the implications and future directions section*.*

Future studies should include a more expansive set of independent variables, based on existing literature in other health professions, to fully understand what factors drive PROM usage by chiropractors for the management of the LBP. Finally, our findings are limited to Australian chiropractors and based on a small selection of LBP PROMs, and so our results cannot be generalised to other countries, body regions/conditions, or PROMs not used in this study.

## Conclusion

Principal chiropractors are more likely to frequently use pain-related PROMs for the management of patients with LBP when compared to associate chiropractors. Although chiropractors place high value on the importance of PROMs for the management of LBP, these beliefs do not translate to increased frequency of PROM usage. Factors, including age, sex, and experience level, also appear to have little influence on PROM usage.

## Data Availability

Not applicable.

## Abbreviations

ACA: Australian chiropractors association
Ahpra: Australian health practitioner registration agency
CAA: Chiropractic association of Australia
CA: Chiropractic Australia
CQU: Central Queensland University
LBP: Low back pain
PROM: Patient reported outcome measure
PROMs: Patient reported outcome measures
